# RETRACTED: Ren et al. Glycine Nano-Selenium Enhances Immunoglobulin and Cytokine Production in Mice Immunized with H9N2 Avian Influenza Virus Vaccine. *Int. J. Mol. Sci.* 2022, *23*, 7914

**DOI:** 10.3390/ijms27167378

**Published:** 2026-08-18

**Authors:** Zhihua Ren, Samuel Kumi Okyere, Ming Zhang, Xin Zhang, Hongxuan He, Yanchun Hu

**Affiliations:** 1 Key Laboratory of Animal Diseases and Environmental Hazards of Sichuan Province, College of Veterinary Medicine, Sichuan Agricultural University, Chengdu 611130, China; zhihua_ren@126.com (Z.R.); 18227592022@163.com (M.Z.); zora0818xin@163.com (X.Z.); 2 Institute of Zoology, Chinese Academy of Sciences, Beijing 100101, China; hehx@ioz.ac.cn

The Journal retracts the article “Glycine Nano-Selenium Enhances Immunoglobulin and Cytokine Production in Mice Immunized with H9N2 Avian Influenza Virus Vaccine” [1] cited above.

Following publication, concerns were brought to the attention of the Editorial Office regarding the integrity of the figures presented in this article [1].

In accordance with the Journal’s standard procedures, the Editorial Office and Editorial Board conducted an investigation, which identified duplicated panels in Figures 1, 2, 6, 7. Although the authors cooperated with the investigation and proposed a post-publication correction, the Editorial Board determined that the identified concerns could not be adequately addressed through a correction.

As a consequence, the Editorial Board decided to retract this publication in accordance with MDPI’s retraction policy.

This retraction was approved by the Editor-in-Chief of the Journal *IJMS*.

The authors have been informed of this retraction and have indicated their disagreement with this decision.

## References

[1] Ren Z., Okyere S.K., Zhang M., Zhang X., He H., Hu Y. (2022). RETRACTED: Glycine Nano-Selenium Enhances Immunoglobulin and Cytokine Production in Mice Immunized with H9N2 Avian Influenza Virus Vaccine. Int. J. Mol. Sci..

